# A shared framework for the common mental disorders and Non-Communicable Disease: key considerations for disease prevention and control

**DOI:** 10.1186/s12888-015-0394-0

**Published:** 2015-02-05

**Authors:** Adrienne O’Neil, Felice N Jacka, Shae E Quirk, Fiona Cocker, C Barr Taylor, Brian Oldenburg, Michael Berk

**Affiliations:** 1 IMPACT Strategic Research Centre, Deakin University and Barwon Health, Po Box 281, Geelong, VIC 3220 Australia; 2 School of Public Health and Preventive Medicine, Monash University, Melbourne, Australia; 3 Department of Psychiatry and Behavioral Medicine, Stanford University, Palo Alto, USA; 4 Centre for Adolescent Health, Murdoch Children’s Research Institute, Melbourne, Australia; 5 Department of Psychiatry, University of Melbourne, Parkville, Australia; 6 Black Dog Institute, Sydney, Australia; 7 Melbourne School of Population & Global Health, University of Melbourne, Melbourne, Australia; 8 Orygen, The National Centre of Excellence in Youth Mental Health, University of Melbourne, Melbourne, Australia; 9 Florey Institute for Neuroscience and Mental Health, Melbourne, Australia

**Keywords:** Non-Communicable Disease, Common mental disorders, Prevention, Depression, Anxiety, Cardiovascular disease, Type 2 diabetes mellitus, Co-morbidity

## Abstract

**Background:**

Historically, the focus of Non Communicable Disease (NCD) prevention and control has been cardiovascular disease (CVD), type 2 diabetes mellitus (T2DM), cancer and chronic respiratory diseases. Collectively, these account for more deaths than any other NCDs. Despite recent calls to include the common mental disorders (CMDs) of depression and anxiety under the NCD umbrella, prevention and control of these CMDs remain largely separate and independent.

**Discussion:**

In order to address this gap, we apply a framework recently proposed by the Centers for Disease Control with three overarching objectives: (1) to obtain better scientific information through surveillance, epidemiology, and prevention research; (2) to disseminate this information to appropriate audiences through communication and education; and (3) to translate this information into action through programs, policies, and systems. We conclude that a shared framework of this type is warranted, but also identify opportunities within each objective to advance this agenda and consider the potential benefits of this approach that may exist beyond the health care system.

## Background

The World Health Organization (WHO) defines Non-Communicable Diseases (NCDs) as medical conditions that cannot be transmitted. They are primarily characterized by chronicity of at least 3-months and their progressive nature [1]. While this definition encapsulates a host of medical conditions, the focus of NCD prevention and control has largely remained on the four conditions that, when combined, account for more deaths than any other: cardiovascular disease (CVD), type 2 diabetes mellitus (T2DM), cancer and chronic respiratory diseases. These conditions are known as the “Big Four.” However, there have been increasing calls to expand the NCD umbrella to include the common mental disorders (CMDs) of depression and anxiety [2,3]. While some have further proposed the inclusion of severe mental disorders (e.g. dementia, schizophrenia and bipolar; the latter two of particular significance given evidence of their shared risk factors, pathways and co-morbidity with NCDs and CMDs), the focus of this paper will be confined to the CMDs, due to their major contribution to the global disease burden. Indeed, the WHO’s Global Action Plan (2013–20) to reduce the global burden of NCDs and preventable mortality now incorporates mental disorder prevention and control targets [4]. However, efforts to curb mental and physical disease still remain largely separate and independent of one another. In an attempt to close this gap, the Centers of Disease Control and Prevention [5] has recently released a public health action plan that aims to integrate mental health promotion and mental illness prevention with chronic disease prevention (see Table 1). Briefly, this framework comprises eight strategies with three overarching objectives: (1) to obtain better scientific information through surveillance, epidemiology, and prevention research; (2) to disseminate this information to appropriate audiences through communication and education; and (3) to translate this information into action through programs, policies, and systems. With a specific focus on the CMDs, this paper argues that implementation of this shared framework is warranted, but also aims to identify opportunities within each objective to advance this agenda. These include: i) greater intellectual and financial investment in research focused on the primary prevention of CMDs as a key strategy for minimizing subsequent NCD risk by delaying or preventing its onset; ii) re-conceptualising the discourse around CMDs to reflect their symbiotic relationship with the NCDs; iii) appropriate education of health professionals, patients and stakeholders; and iv) the integration of interventions and programs that consider context, cost and responsibility. We conclude by considering the potential benefits of this approach beyond the health care system.

**Table 1:** **Eight framework components of the CDC**

	Description of framework objectives
1.	Surveillance to develop shared operational definitions and determinants of mental illness
2.	Epidemiological research into determinants and protective factors for mental health and their relationships to chronic diseases
3.	Prevention research to determine the importance of mental illness/health as factors in public health promotion and prevention
4.	Communication to develop culturally, linguistically, and developmentally appropriate educational products
5.	Education of Health Professionals including the development of appropriate education plans for professional audiences
6.	Program Integration Support at various jurisdictional levels
7.	Policy Integration that involves the development of policies at all government levels for all audiences and stakeholders
8.	Systems to Promote Integration such as establish systems integration to promote program and policy integration across multiple infrastructures

### Is a shared framework for CMD and NCD prevention and control warranted?

The statement there is “no health without mental health” [6] has been globally endorsed by authoritative health bodies. Indeed, we have previously argued this adage should be reflected within the NCD umbrella by including CMDs for three key reasons. First, when the global burden of disease is viewed in terms of disability, and not death, major depression is the second leading cause of disability. By comparison, the Big 4 account for only half (54%) of all NCD-related disability adjusted life years (DALYs). A recent report suggests the annual cost of depression is $200 billion in the United States alone [7], which is compounded by the impact of anxiety disorders that often co-occur. Anxiety is the most common mental illness in the United States, affecting 40 million individuals [8]. Second, from an etiological perspective, CMDs and NCDs are strongly inter-connected, highly co-morbid and share important pathways to disease. It is well established those with a mental disorder are more likely to have a medical condition [9], while individuals with a somatic condition such as T2DM, are at increased risk of a mental disorder such as depression [10] or anxiety [11]. For example, mental disorders and CVD, the two dominant contributors to the global economic burden of NCDs [12], share a close relationship. The presence of a clinical depressive disorder elevates the risk of incident CVD [13]. A meta-analysis also revealed individuals with anxiety disorders are at increased risk of incident CVD and cardiac death [14]. In addition to mental disorders, specific depressive and anxiety sub-types contribute to adverse cardiac-related outcomes. Somatic subtypes of anxiety disorder can predict coronary heart disease (CHD) while cognitive subtypes contribute to in-hospital cardiac complications in acute coronary syndrome (ACS) patients, independently of depression [15]. The converse is also true; a cardiac event elevates a patient’s risk of developing depression and anxiety. Thus, the American Heart Association recently recognized depression as a risk factor for poor prognosis following a cardiac event [16]. Similar bi-directional associations exist for depression and T2DM [17]. These conditions likely share a common diathesis whereby the vulnerability factors for depression and anxiety can contribute to the onset of other disorders. Their mutually reinforcing nature may lead to a progressive cycle of psychological and physical ill health across the life course [18]. Third, there is accumulating evidence that the same four lifestyle risk factors, poor diet, physical inactivity, smoking and alcohol use, also contribute to the onset and trajectory of the CMDs in some important ways (explored in further detail in the next section). These risk factors are likely to cluster and further exacerbate the risk of other medical conditions associated with poor mental health. While beyond the scope of this paper, the same is true for some of the serious mental illnesses such as schizophrenia and bipolar disorder. In these, diet [19], smoking [20], substance and alcohol abuse and physical inactivity [21] adversely modify risk or prognosis. Risk factor clustering is more common among people with a CMD than in the non-CMD population [22]; for example, alcohol consumption is closely associated with smoking, obesity and poor diet in depressed populations [23]. Indeed, the potency of these risk factors is likely to be multiplicative as they interact to increase the risk of CMD onset. An individual’s susceptibility to a particular risk factor, and its magnitude of effect on disease progression, will also differ according to their risk factor profile and the weighting of each risk factor. For example, while poor lifestyle choices such as smoking may be independent risk factors for CMDs [24], its effects are likely to be pronounced in those pre-disposed to CMDs by genetic or other environmental factors.

Shared risk factors and the high prevalence of co-morbidity between NCDs and CMDs provide clear support for the implementation of a shared framework for the prevention and treatment of NCDs and CMDs. Within the CDC’s three framework objectives, we now consider key ways in which this agenda could be advanced.

#### Obtain better scientific information: Greater intellectual and financial investment into research specifically around the primary prevention of CMDs as a key strategy for minimizing subsequent NCD risk by delaying or preventing its onset

Many of the greatest victories in oncology and cardiology have come through prevention rather than treatment advances. Public health policies around smoking, and risk factor surveillance such as dyslipidaemia, hypertension and pap smear tests being notable exemplars. When compared with other disciplines of medicine, psychiatry has arguably lagged behind in the field of prevention science. Not withstanding recent indications that diet, vitamin D, smoking and exercise may be important factors in the onset of the CMDs (e.g. [25]), there remains a clear need identify and quantify plastic risk factors that are germane to psychiatric disorders. Large-scale, population-based studies with the power to calculate the population attributable risk of specific CMDs from modifiable risk factors (akin to the INTERHEART (2003) study for myocardial infarction [26]) are critical for guiding population-wide NCD prevention and control strategies, particularly for the primary prevention of CMD. These data could also guide the development of equivalent risk factor equations, assessment tools and treatment algorithms for CMD prevention that are often used in other areas of medicine for guiding investment and resource allocation. While some have attempted to develop risk algorithms based on individual and collective risk factors to predict future risk of depression and anxiety [27], they have not been developed based on international data and, despite demonstrating sound psychometric properties, are not routinely used in clinical practice. The development and dissemination of such materials could aid physicians and public health practitioners in a range of health care settings to identify those at high risk of CMD. Similarly, given the well-established and respective links between depression and incident CHD and T2DM, for example, widely used risk assessment tools such as the Framingham Risk Equation (for 10 year CVD risk) and AUSDRISK (for 5-year diabetes mellitus risk) could be updated to include depression parameters.

Explication of the shared biological pathways underpinning the risk for commonly-comorbid psychological and somatic illness will greatly advance the ability to develop effective preventive strategies for both. For example, immune system dysfunction is a common feature of virtually all NCDs as well as depression. Related to this understanding is the new knowledge regarding the human gut microbiome as the core driver of immune functioning, as well as the development of the brain, metabolic and innate immune system in early life. Indeed, there is emerging evidence to suggest that the main environmental risk factors implicated in the increasing prevalence of NCDs, ie. poor diet and sedentary behavior, mediate their effects through immune pathways, with downstream effects on insulin resistance, obesity, cardiovascular disease, as well as mood and behavior. New insights in this rapidly developing field point to the utility of taking population-level primary prevention approaches to both NCDs and CMDs.

#### Disseminate the information to appropriate audiences: Re-conceptualize discourse around CMDs within the context of NCDs and educate health professionals, patients and stakeholders accordingly

There is little doubt the way in which key stakeholders from health professionals, industry, government to patients, conceptualise CMDs and NCDs, is vast; there is a spectrum over which these conditions can be viewed. Using the example of diabetes and depression, Fisher et al. (2012) describes the different ways of conceptualising NCDs and CMDs for prevention and treatment: as “a) categories or dimensions; b) single problems or parts of broader categories, e.g., metabolic/cardiovascular abnormalities or negative emotions; c) separate comorbidities or integrated so that depression is seen as part of the comprehensive, normal clinical picture of diabetes; and d) expressions of a shared, complex biosocial propensity to chronic disease and psychological distress” [18]. He subsequently argues successful models of interventions should reflect this, and the commonalities among chronic mental and physical disorders [18].

Such integrated models of care already exist. For example, a key component of the IMPACT model is the education and upskilling of health care professionals to identify, appropriately refer and communicate with patients about symptoms of CMDs. This approach has largely been used for the concurrent management of depression, CHD and T2DM in primary care settings [28] and has shown to be more effective and cost-effective for chronic disease management than standard care. Practice Nurses are educated to act as case managers who aim to provide continuity of care. They are trained to: encourage support and effective communication to patients from clinicians, utilizing evidence-based guidelines to promote patient self-management, monitoring risk factors, scheduling visits and providing audit information [29]. However, such models however have been subject to criticism.

For example, some have argued that integrated care models have limited generalizability, require a cultural shift in norms and roles, are too resource intensive to implement, and create unnecessary overlap with other related programs [30]. However, there are data that refute some of these suggested barriers. Rollman et al. has shown a similar ‘upskilling’ model when enacted over the telephone to be generalizable to more specialized populations. The Bypassing the Blues study found this model efficacious for reducing depression in patients undergoing coronary artery bypass grafting surgery [31]. Further, others have shown it to be scalable. Using a cluster-randomised design in general practices across Australia, the TrueBlue study provided evidence that with sufficient training and ongoing support such as planning tools and peer support, upskilling and educating Practice Nurses can be effective for the management of co-morbid depression, CHD and/or diabetes as well as fostering a therapeutic alliance with (and education) of patients [32]. Indeed, the education of students, graduates and health professionals and stakeholders on evidence based programs in this area is required to perpetuate a culture that re-conceptualises the idea that CMDs and NCDs are largely individual conditions. It is acknowledged however that the scalability of models such as the aforementioned remain dependent largely on local culture, health systems, workforce and many other contextual factors and influences; all of which affect the adaptation of these types of models within real world settings.

#### Translate the information into action: Develop appropriate interventions and prevention programs considering context, cost, responsibility and translation

An integrated framework that considers both CMDs and NCDs needs to operate within existing structural, organizational, cultural and economic barriers within existing health care systems. For example, while case management systems have long been used in primary care settings to promote risk factor reduction in patients with NCDs, arguably the most important characteristic underpinning collaborative care models such as IMPACT is its ability to capitalise on existing workforce and financial infrastructures by refocussing the roles of the existing members and organisational structures of the setting. Fisher et al. [3] maintains that failure to consider these contextual factors can result in a number of potentially deleterious outcomes. First, efficacy and/or effectiveness of specific interventions may be diluted. Second, the benefits of interventions may be further underestimated should contextual moderators remain unaccounted for in analyses. Third, individuals participating in these interventions could be perceived as responsible for their condition resulting in a type of ‘victim blaming’ [3]. To this end, future opportunities exist to explore and evaluate different approaches to increase awareness and understanding of these issues in both health professionals and patient populations.

Currently, the health care system is faced with significant and increasing pressure including the demands of a higher prevalence of CMD [32] and NCDs which is further compounded by other important factors like the obesity and metabolic syndrome epidemics. Some have argued that, despite a long and complicated history, continuing to regard CMDs as an independent health domain, with siloed budgets and services, perpetuates the notion that mental health investment has unaffordable opportunity costs [3]. This points to the critical need to explore shared and more efficient approaches to CMD treatment and widen the current focus on treatment of CMDs to give equal weight to prevention strategies; many of which overlap with NCD prevention. However, the perceived cost associated with the primary prevention of diseases, much less that of CMDs, remains a barrier to investment in this area. A WHO report suggests a key factor underscoring the traditional focus on, and preference for, curative strategies are the short-term, tangible benefits as opposed to the longer pay-off periods that are required to see the effects of prevention [31]. This is particularly evident as the cost of healthcare increases globally, thereby increasing competition for resources [31].

### Where to from here: translation into policy and practice?

From a policy perspective, a trans-disciplinary effort is required from both the key stakeholders within the mental health, public health and medical sector to provide a collective voice if public policy in this arena is to be successfully changed. Currently, policy development in areas that are known to impact lifestyle behaviours (e.g. food policy, taxation) in countries such as Australia is heavily influenced by industry and the business sector. To this end, the voice of mental health advocates becomes more important for promoting the shared framework agenda. Practically, a greater focus on the integration of mental health research into services, leverage from existing infrastructure, lobbying for greater financial investment for research and design and a greater input on mental health outcomes within medicine may facilitate better availability and translation of research findings around prevention and control of CMDs to the policy arena.

### The benefits of a shared approach to CMD and NCD: beyond the health care system

The wellbeing and health benefits resulting from investment in prevention science is of clear economic importance [33], particularly considering the associated costs largely attributed to increased health care use and expenditure (e.g., specialist care, hospital care and medication) and lost productive time. A substantial economic burden from NCDs is borne by workplaces, in particular, the loss in productivity resulting from sickness absence and continuing to work when ill, a behavior known as presenteeism [34]. Additionally, a sizeable minority of employed people with NCDs, an estimated 18% in Australian employees, will also be managing another major health conditions including heart disease, arthritis, depression, asthma, and diabetes [34]; multimorbid disease combinations which commonly include depression [35]. Further, data demonstrate absenteeism is higher amongst people with one or more chronic illnesses, employees with a chronic illness have higher rates of long-term work-disability than the general population [36], and, as one recent Australian study demonstrated co-morbid psychological distress causes an increased risk of productivity loss, from both absenteeism and presenteeism, for a range of health conditions. Therefore, with an ageing global workforce, a greater understanding of how to promote the health, continued workforce participation and subsequent productivity of workers with multiple NCDs is a priority, as the impact of multimorbidity is not restricted to older adults outside the working population.

To date, most of the research on multimorbidity and health and social outcomes has focused on quality of life [37], use of medical services [38], and hospitalisation and mortality [39]. Further, the majority of research has been conducted within clinical or primary care [35,40], or selected older adult populations [40]. Given that many NCDs affect healthier, working age adults [34], this focus on clinical populations, and clinical outcomes, provides insufficient evidence to inform public health approaches to improving the health of the workforce [35,41]. Therefore, increased focus needs to be on developing: i) evidence-based guidance to inform a reduction of the burden of common NCDs and co-morbid CMDs among working age adults; ii) the prevention of long-term work absences and the associated costs and generally poor outcomes of return-to-work programmes and; iii) the development of decision aids for individuals, their employers and their clinicians to help guide management of work demands and work attendance.

## Conclusion

There is a need to close the gap between the epidemic growth of CMDs, like depression and anxiety, and NCDs, such as type 2 diabetes and cardiovascular diseases, and the provision of appropriate healthcare and public health programs that address the shared determinants and pathways of these disorders. A shared framework for disease prevention and control appears warranted, yet there remains a range of contextual and economic factors to consider for wide-scale implementation in real world settings. Specific considerations include the responsibility of investment and cost recovery, and better education of key stakeholders. Intellectual and financial investment in research remains an imperative, in order to advance our understanding of the epidemiology, etiology and determinants of mental conditions both independently and as they relate to physical disorders.
