# Correction: A shared framework for the common mental disorders and Non-Communicable Disease: key considerations for disease prevention and control

**DOI:** 10.1186/s12888-022-03991-3

**Published:** 2022-05-27

**Authors:** Adrienne O’Neil, Felice N. Jacka, Shae E. Quirk, Fiona Cocker, C. Barr Taylor, Brian Oldenburg, Michael Berk

**Affiliations:** 1 IMPACT Strategic Research Centre, Deakin University and Barwon Health, Po Box 281, Geelong, VIC 3220 Australia; 2 School of Public Health and Preventive Medicine, Monash University, Melbourne, Australia; 3 Department of Psychiatry and Behavioral Medicine, Stanford University, Palo Alto, USA; 4 Centre for Adolescent Health, Murdoch Children’s Research Institute, Melbourne, Australia; 5 Department of Psychiatry, University of Melbourne, Parkville, Australia; 6 Black Dog Institute, Sydney, Australia; 7 Melbourne School of Population & Global Health, University of Melbourne, Melbourne, Australia; 8 Orygen, The National Centre of Excellence in Youth Mental Health, University of Melbourne, Melbourne, Australia; 9 Florey Institute for Neuroscience and Mental Health, Melbourne, Australia

**Correction: BMC Psychiatry 15, 15 (2015).**

**https://doi.org/10.1186/s12888-015-0394-0**

Following publication of the original article [1], the authors identified a missing citation under the heading Background. The added reference is given below.

6. Oldenburg, B., OʼNeil, A., & Cocker, F. (2015). Public health perspectives on the co-occurrence of noncommunicable diseases and common mental disorders. In Comorbidity of mental and physical disorders (Vol. 179, pp. 15–22). Karger Publishers.

The original article [1] has been corrected.
